# Indole Content Profiling During Biological Ageing of Cava Sparkling Wine

**DOI:** 10.3390/foods14050722

**Published:** 2025-02-20

**Authors:** Clara Abarca-Rivas, Alba Martín-García, Montserrat Riu-Aumatell, Elvira López-Tamames

**Affiliations:** 1 Polyphenol Research Group, Department of Nutrition, Food Science and Gastronomy, XIA, Faculty of Pharmacy and Food Sciences, Institute of Nutrition and Food Safety (INSA-UB), CCNIEC Research Group “Antioxidants Naturals: Polifenols”, University of Barcelona, 08028 Barcelona, Spain; claraabarca@ub.edu; 2 Aroma, and Food Quality Factors Group, Department of Nutrition, Food Science and Gastronomy, XIA, Faculty of Pharmacy and Food Sciences, Institute of Nutrition and Food Safety (INSA-UB), CCNIEC Research Group “Aroma and Food Quality Factors Group”, University of Barcelona, 08028 Barcelona, Spain; albamart3@gmail.com (A.M.-G.); e.lopez.tamames@ub.edu (E.L.-T.)

**Keywords:** melatonin, sparkling wine, indole, tryptophan ethyl ester, UHPLC-MS/MS

## Abstract

Indoles are bioactive components found in wine products and are associated with yeast activity. Cava, a Spanish sparkling wine, is characterized by aging in contact with lees, making it a potential matrix for indoles. Therefore, the aim of this study was to determine the indole content in Cava produced at an industrial scale. Nine indoles were analysed by Ultra High-Performance Liquid Chromatography—Tandem Mass Spectrometry in Cava samples with different ageing times (n = 74). Significant amounts of tryptophan (2.3–1680.4 μg/L), tryptophan ethyl ester (0.1–5.2 μg/L), 5-methoxytryptophol (0.3–29.2 µg/L) and n-acetyl serotonin (0.3–2.3 μg/L) were determined. Tryptophan and tryptophan ethyl ester were positively correlated and decreased with ageing time. In fact, a concentration of less than 0.56 μg of the latter indole can become a marker of the most aged Cavas. The ageing time in contact with lees seems to play a key role affecting the indole content, since base wines show high amount of tryptophan and tryptophan ethyl ester while aged sparkling wines have values around the lower 95% confidence limit. Notably, the identification of tryptophan ethyl ester as a potential marker for aging in Cava suggests a new avenue for further research and quality assessment in its production.

## 1. Introduction

Melatonin (MEL), an indole (N-acetyl-5-methoxytryptamine) secreted mainly by the pineal gland and by plants as a secondary metabolite [1,2], has been reported as a bioactive compound that presents positive effects on human health [3,4]. These health benefits are attributed to its antioxidant, anticarcinogenic and neuroprotective properties [5,6]. MEL’s antioxidant capacity has been confirmed in in vivo studies by mitigating chronic age-related oxidative stress [7] and reducing blood pressure in men with chronic hypertension [8]. Clinically, MEL effectively treats sleep disorders by restoring circadian rhythm, which especially benefits people with neurodegenerative diseases [4]. Moreover, the European Food Safety Authority (EFSA) accepted scientific evidence as a health claim when dosed between 0.5 and 5 mg [9].

Melatonin is naturally present in some foods from the Mediterranean Diet. Grapes and olive oil were the first products of this diet in which melatonin was detected [10]. It was first identified in wines in 2006 [1,2]. Although in wine MEL has been detected at levels ranging from 0.005 ng/mL to 1.50 ng/mL [11,12], Rodriguez-Naranjo et al. [13] found significantly high concentrations (245–423 ng/mL) in ten different single-varietal wines.

In addition to MEL, its derivatives and precursors, such as tryptophan (TRP) and tryptophan ethyl ester (TEE) have also been detected [14]. Moreover, wine can contain other tryptophan derivatives whose biological properties are not fully understood yet and further research is needed to uncover the specific characteristics and functions of these compounds [14,15]. Interestingly, TEE is one of the predominant tryptophan derivatives in wine, surpassing the concentration of MEL [16].

**Figure 1:**
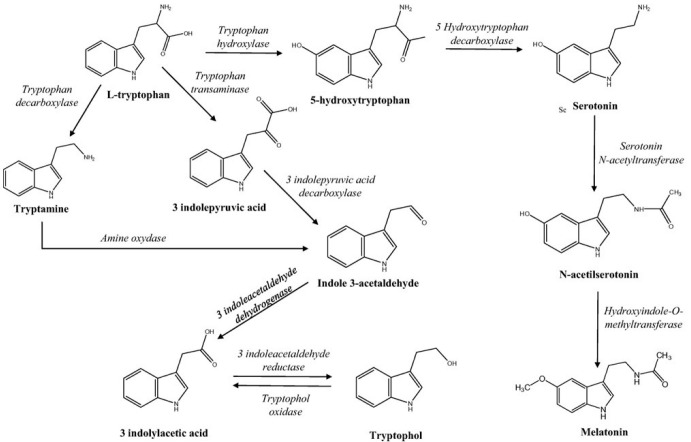
Metabolism of indole compounds derived from l-TRP [16].

MEL is synthesized from TRP, having as intermediary compounds 5-hydroxytryptophan (5-OHTRP), serotonin (SER), and ultimately N-acetylserotonin (NSER). MEL can be metabolized by deacetylation to 5-methoxytryptamine as can be seen in Figure 1 [2,17]. In addition, MEL can similarly be produced by O-serotonin methylation followed by N-methoxytryptamine acetylation in the yeast cell (Figure 1) [13].

In fact, it has been reported that during the winemaking process melatonin is produced after the yeast inoculation [2]. Therefore, the role of *Saccharomyces cerevisiae* has been shown to play a key role regarding the melatonin content and its isomers in wine [11,17]. During alcoholic fermentation, most yeast strains synthesize TRP and different indole compounds derived from it. In addition, yeast can also produce 5-hydroxytryptophan (5-OHTRP), an intermediate from MEL metabolism, through different pathways [16]. For this reason, among all the factors affecting melatonin content, yeasts are supposed to be the most important [18].

Several techniques have been used for the determination of MEL in foods, such as liquid chromatography (LC) coupled with different detectors, gas chromatography (GC) related methods, or capillary electrophoresis (CE) methods [19]. In case of wine, techniques such as LC-tandem mass spectrometry (MS/MS), Ultra High-Performance Liquid Chromatography (UHPLC)-MS/MS, or LC coupled with high resolution mass spectrometry (HRMS) have been widely employed [19,20,21]. However, these techniques were not optimized for sparkling wines. Therefore, we previously optimized and validated the UHPLC-MS/MS method [22].

One type of wine where these indoles may be present is Cava, which is a sparkling wine produced in Spain (Catalonia). Cava is characterised by a two-stage fermentation process: a first fermentation of the must to elaborate the base wine and a second fermentation performed in the same bottle that reaches to the consumer. Since Cava is a quality wine produced in a specific region (QWPSR), the regulation establishes that the wine must undergo a biological ageing process where the yeast lees from the second fermentation remain in contact with the wine in the same bottle where the fermentation takes place [23]. In fact, the biological ageing time varies, but in the case of high-quality sparkling wines from Europe, such as Cava or Champagne, it ranges from a few months to several years. Specifically, Cava is aged for a minimum of 9 months, Reserva category wines for a minimum of 15 months, and Gran Reserva for a minimum of 30 months [23]. During the vinification of sparkling wine, amino acids such as TRP are released from the yeasts into the wine [24], which contribute to the development of the characteristic flavor and aroma of sparkling wines [22]. Therefore, Cava may be a good matrix to contain indoles. To our current knowledge, there are no studies analyzing the effect of biological ageing on the content of melatonin and its related tryptophan-derived compounds in this kind of sparkling wine.

In view of the above, the aim of the present study was to evaluate the content of indoles in Cava and the impact of the aging time on them. Hence, it could help to clarify the effect of sparkling wine technology on indole content.

## 2. Materials and Methods

### 2.1. Chemicals and Reagents

L-Tryptophan, 5-hydroxy-L-tryptophan, serotonin hydrochloride, N-acetyl-5-hydroxytryptamine, melatonin, 5-methoxy-tryptophol, 5-hydroxyindole-3-acetic acid, 5-methoxy-3-indoleaceticacid, L-tryptophan ethyl ester hydrochloride, L-tryptophan-d5 with an isotopic purity of >97% (indole-d5) (internal standard, IS), hydro-chloric acid, and ammonium hydroxide ACS were supplied by Sigma-Aldrich (St. Louis, MO, USA).

Acetonitrile of LC-MS grade was from Riedel-de Haën (Seelze, Germany), and methanol, hexane, and acetone of HPLC grade were purchased from Merck (Darmstadt, Germany). Ultrapure water was obtained using a Milli-Q system (Millipore, Milford, MA, USA).

The single standard stock solutions and IS solution were prepared weekly by dissolving known amounts of pure standards in methanol (1000 mg/L) and then stored in the dark at 4 °C until analysis. Spiking standard solutions (20 mg/L) were prepared weekly by diluting standard stock solutions with methanol.

### 2.2. Vineyard Conditions

The vineyards from which the study samples were taken have a calcareous soil, which provides good drainage and moisture retention. Climatic conditions during the grape growing season were characterised by warm temperatures (30 °C on average), moderate humidity and seasonal rainfall patterns (500 mm rainfall average) and dry conditions in summer that favor grape ripening. Grapes were produced following standard agricultural practices and quality controls were made in each cellar.

All the samples in the study belong to the Cava DO. This means that all wineries were subjected to quality checks and inspections carried out by the Regulatory Council technical services at different times throughout their production. These checks range from the vineyard, yield control, quality monitoring and the final analysis of the destination of these grapes, without forgetting the repeated inspections throughout the entire Cava production process. The base wines and subsequent Cava samples were elaborated with the usual coupage of Macabeu, Parellada and Xarel·lo grape varieties.

### 2.3. Samples

**Figure 2:**
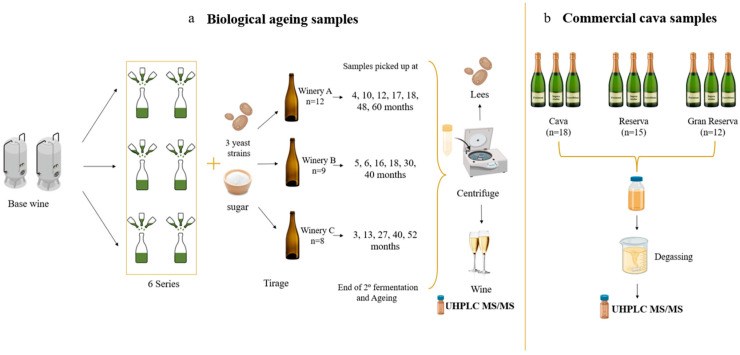
Sampling process and preparation of biological ageing series (**a**) and commercial cava samples (**b**).

Commercial Cava and Cava samples produced at industrial scale were analysed (Figure 2). For the biological ageing series (Figure 2a), Cava samples were produced employing the traditional method and collected during the ageing process from Freixenet, Segura Viudas and Castellblanch wineries (Catalonia, Spain).

For the second fermentation in the bottle from the base wines, 40–42 mL of tirage liqueur was added. This was composed of 1–2 × 10^6^ yeast cells/mL, 500 g sucrose, 0.1–0.2 g bentonite and 50 mL/hL ammonium phosphate (to stimulate yeast growth). The yeast employed belongs to the private collection of the three wineries.

Each winery provided samples with different ageing time ranging between 3 and 60 months (Figure 2a). For each sample period, four bottles of each biological aged wine were opened and frozen. The remaining bottles were stored in the winery conditions until the next sampling time. There was a total of 6 base wines and 23 Cava of different category collected from the wineries: 5 were post-fermentative samples (3, 4, 5, and 6 months), 4 Cava (10, 12, 13 months), 8 Reserva (17, 18, 27, 30, 40 months) and 6 Gran Reserva (30, 40, 48, 52, 60 months).

In relation to the commercial samples (Figure 2b), 45 Cava were purchased from local supermarkets corresponding to different regions of Catalonia (Spain) included in the DO Cava as well as from different classifications according to their biological ageing in contact with lees: 18 samples of Cava, 15 were Reserva and 18 labelled as Gran Reserva. In the commercial samples, there was more variability as all the authorized varieties were used. Most of the samples of both commercial and winery Cavas were elaborated using white grape varieties (Macabeu, Parellada and Xarel·lo) (n = 40), nevertheless, 5 samples used red grapes (Pinot noir, Trepat, Monastrell and Garnacha) to elaborate rosé Cava. As commercial sparkling wines that had been produced on an industrial scale at different wineries, each one used different yeast strains from its private collection.

### 2.4. UHPLC-MS/MS Conditions

A total of 9 indoles: 5-methoxytryptophol (5MTL), TRP, TEE, NSER, MEL, SER, 5-OHTRP, 5-hydroxyindolacetic acid (5OHIA) and 5-methoxyindolacetic acid (5MIA) were determined during biological ageing and analysed in different real Cavas (n = 74) produced at industrial scale according to their commercial category (Cava, Reserva and Gran Reserva).

Biological samples were centrifuged to eliminate yeast lees and commercial samples were degassed by magnetic stirring for 5 min. These samples were stored in 250 mL amber bottles at −20 °C until analysis and, prior to analysis, filtered with a nylon membrane filter (0.2 μm) (Waters, Milford, MA, USA). To avoid degradation of melatonin, all sample preparation was carried out under reduced light conditions.

The chromatography analysis was performed following the method previously described and validated [22]. Briefly, an Acquity system equipped with a binary pump system (Waters, Milford, MA, USA) was used for sample analysis by UHPLC-MS/MS. Detection was performed using a triple quadrupole mass analyser (TQD) (Waters, Milford, MA, USA). The column used for the analysis was a BEH C18 Shield (150 × 1.0 mm i.d.) with a particle size of 1.7 μm (Waters, Milford, MA, USA) which was maintained at 30 °C. Chromatography conditions were as follows: elution was in linear gradient from 95% of mobile phase A (5:94.9:0.1 *v*/*v*/*v* ACN/water/formic acid) and 5% of B (95:4.9:0.1 *v*/*v* ACN/water/formic acid) to 25% of A in 8 min, with a flow rate of 0.125 mL/min. Samples of 8 μL injected and kept at 10 °C. Between each injection, the system was stabilised for 1 min at the initial conditions and a standard washing procedure with mobile phases A and B was included. At the start of a new batch of samples, it was checked for carry-over by injecting water (blank sample) after a spiked sample.

The electrospray interface (ESI) was operated in positive mode; the source temperature was fixed at 130 °C, the capillary voltage was set at +3.0 kV, and the desolvation temperature was set at 350 °C. The cone gas (nitrogen) flow rate was 350 L/h. The gas used in the collision cell was argon at a flow rate of 0.1 mL/min.

For quantification, the multiple reaction monitoring (MRM) mode was used in which two specific MRM transitions of the deprotonated molecular ions were monitored for each compound. The product ion with the highest signal was chosen. MassLynx software (version 4.1) was used for data acquisition and integration.

Once the indoles were quantified, a calibration curve was first performed to compensate for random and systematic errors such as non-spectral interferences from matrix components and fluctuations due to the method and instrumental errors. As shown in Tudela et al. [22] the calibration curve exhibited a significant linear relationship between the chromatographic response and the indole concentrations (R^2^ > 0.99), indicating a high precision and accuracy of the analytical method employed). Both repeatability and accuracy were adequate for all the indoles (Table 1).

### 2.5. Statistical Procedures

Data was processed using Prism 9 version 9.5.1 (733) (GraphPadSoftware, LLC., San Diego, CA, USA). Simple and multifactorial ANOVA and the corresponding pairwise post-hoc comparison of the means were conducted using Bonferroni’s correction, with significance level of 0.05. The ageing time, commercial category, titratable acidity, pH and volatile acidity of the samples were considered. Pearson’s correlation coefficients for the parameters were also calculated. Clustering heatmap was used to visualize how the means of different wine categories compare to each other and to reveal patterns and relationships in the data.

## 3. Results and Discussion

### 3.1. Oenological Parameters in Cava Samples

**Figure 3:**
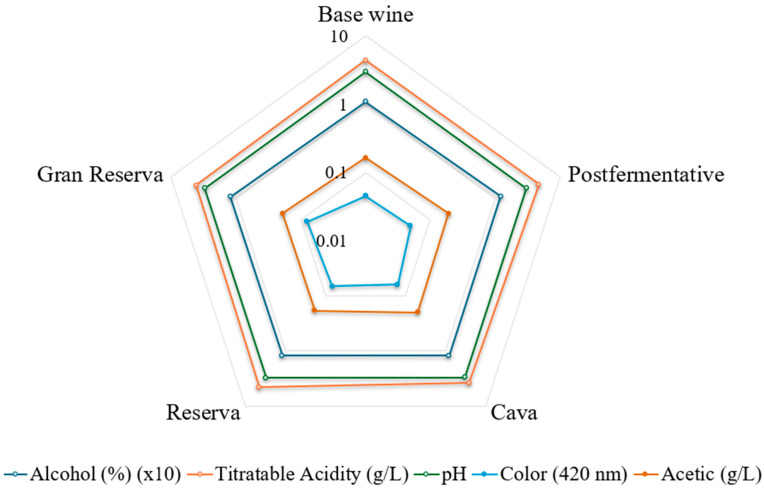
Radar chart of winery samples characterization according to sparkling wine category (logarithmic scale).

In the present study, the 9 indoles were analysed in 74 samples of Cava (29 from wineries and 45 from commercial Cava). In addition to the indole concentrations, data on alcohol content, titratable acidity, pH, and acetic acid were provided for the cellar samples. These oenological parameters were comparable across the different categories of Cava and exhibited no significant differences (Figure 3). The radar chart data has been plotted on a logarithm scale of 10. The alcohol content was previously normalized by a factor of 10 to align it with the scale of the other compounds. On average, the following values were reported: 11.80% alcohol, 4.34 g/L titratable acidity, a pH of 3.01, 0.8 g/L acetic acid and an absorbance of 0.06 at 420 nm.

### 3.2. Indoles in Cava Samples

Of the total of nine indoles analysed, four were detected at levels greater than the limit of detection (LOD) in Cava. However, neither MEL nor its isomers, SER, 5-OHTRP, 5OHIA or 5MIA were found in any sample. The current study did not detect MEL in Cava, which aligns with findings from other researchers who also reported the absence of this compound in wine [14,25,26]. As shown in the literature, MEL content can vary significantly due to several factors, including different grape varieties (ranging from 0.16 to 129.5 ng/g), various agronomic practices, and the yeast strain used [27,28]. It has also been demonstrated that melatonin in wine is primarily synthesized at the beginning of fermentation and subsequently decreases [29,30]. Since the base wine for Cava has already undergone a first alcoholic fermentation, it is consistent that this indole is not present in any of the samples. Therefore, although some grapes and wine are possible sources of melatonin, cava cannot be considered one of them.

As previously stated, MEL has been shown to be synthesized from tryptophan, but it can also be formed by O-methylation of serotonin followed by N-acetylation of 5-methoxytryptamine in the yeast [13]. The lack of detection of MEL levels in Cava wines could be due to the wine-making practices needed for their elaboration, which involved a second fermentation and an extensive ageing process that, in some categories, exceeded a year. These processes could contribute to the transformation and reduction of MEL present in the base wines, thus explaining its absence in the final Cava products. In addition, the lack of MEL can also be related to a higher amount of its isomers, as reported in some wines [25,31]. In the present study, TEE was the isomer found in the Cava samples.

SER, 5-OHTRP, 5-OHIA and 5-MIA indoles were also not detected in Cava samples as seen in some recent studies [26,32]. SER is not commonly found in wines, although according to [33], malolactic fermentation bacteria significantly affect the formation of this compound. The lack of detection of this compound was therefore consistent with the fact that the Cava analysed did not undergo this type of fermentation. The other compounds have only been isolated occasionally, such as 5OHIA, which was detected in an Albanian wine [17]; and 5-OHTRP, which was first reported in the must in 2019 but was found to be rapidly consumed by yeasts during alcoholic fermentation [34].

### 3.3. Indoles Content and Ageing Time

Regarding the indoles detected (5MTL, NSER, TRP, TEE) in the samples, Table 2 shows the concentration present according to the ageing time of winery and commercial samples. Moreover, there was a clear difference in the profile of the different wine categories in terms of indole content.

**Figure 4:**
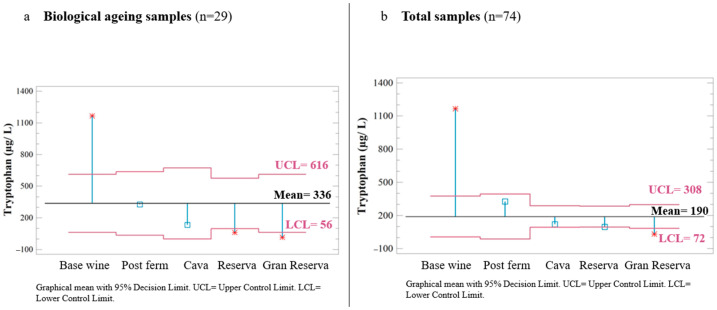
Analysis of the graphical means with 95% decision limits for the TRP μg/L content in biological ageing samples (**a**) and in the total of samples (**b**) by category. UCL: Upper Control Limit and LCL: Lower control limit. * indicates that the mean of each category is significantly different from the mean of the samples.

Overall, the most abundant indole was TRP. It was found in greater quantities in the base wine, and it decreased with the ageing time of Cava. Statistically significant differences were observed in the content of the base wine and Gran Reserva compared to the other categories during biological ageing samples (n = 29) (Figure 4a). Considering the total number of samples (n = 74) (Figure 4b), including commercial Cava, these differences were also shown, but to a lesser extent. This highlights the influence of lees on the content of this indole, among other changes they can exhort.

This was in line with what has been reported in some commercial wines of different varieties, where the amount of TRP decreased during ageing [35,36,37]. The decrease of TRP could probably be because *S. cerevisiae*, consumed TRP for their growth [16] before ageing in contact with lees. Although there is great variability in TRP content (2.3 to 1680.4 μg/L), it should be noted that for all Cava samples under the Gran Reserva category, the average content was below 55.4 μg/L. Moreover, Baenas et al. [38] reported that lees by products have greater concentrations of compounds derived from tryptophan compared to TRP itself.

Another compound present in all samples was TEE. The relationship between melatonin concentrations and this isomer is not known but it appears that they come from different biosynthetic pathways, possibly TEE coming directly from TRP [14,19]. It is the most abundant putative melatonin isomer detected in red wine and has the same molecular weight as melatonin. This compound is relevant for its possible nutritional role, as it could provide a tryptophan reserve capable of crossing the gastrointestinal tract and possibly the blood-brain barrier [39].

In the present study, TEE was detected in amounts ranging from 0.1 to 5.2 μg/L (Table 2), presenting significant differences among all categories. Notably, all Gran Reserva Cava samples, both commercial and biological aging, exhibited TEE concentrations below the threshold of 0.56 μg TEE/L. This threshold may therefore serve as a potential marker to distinguish Gran Reserva Cava (aged for a minimum of 30 months) from other Cava categories. Cava with TEE levels above 0.56 µg/L would be categorized as either Cava (aged for a minimum of 9 months) or Reserva (aged for a minimum of 15 months), pending further investigation. This finding suggests a new avenue for further research and quality assessment in Cava production.

**Figure 5:**
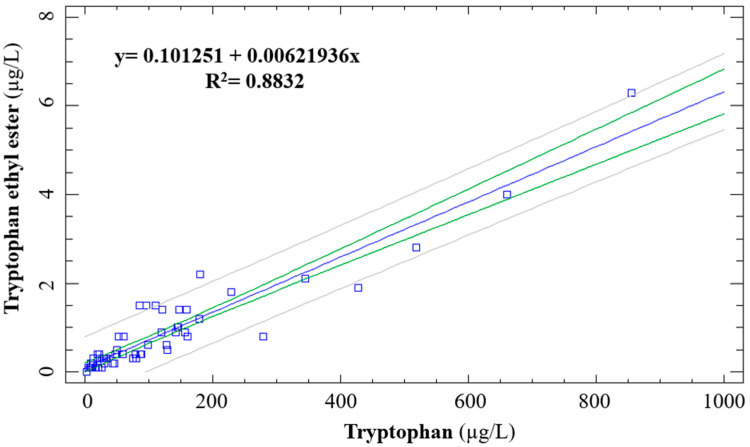
Correlation between TRP and TEE in commercial and winery samples.

Moreover, both TRP and TEE decreased with ageing time, showing an exponential trend line. In fact, these similar trends were reinforced by the fact that these two indoles were significantly correlated (*p* < 0.001) (Figure 5). According to the equation of the correlation line (Figure 5), a linear relationship was shown between the TRP and TEE which reflected a moderate positive association between the two variables. Furthermore, the coefficient of determination (R^2^) indicated that 88.32% of the variability in the variable y can be explained by the variability in the variable x through the regression line. Although Iriti et al. [14] found this correlation during alcoholic fermentation of grape must, to the best of our knowledge, this is the first time that it has been reported during biological ageing.

Another indole studied was NSER, which is an intermediate metabolite in melatonin synthesis that also has been attributed with health-promoting properties such as a neuroprotective effect [40]. NSER, it has just been found in wines in a couple of studies showing contradictory results [34,41]. In one of them, it only appeared in a very small amount in the must and disappeared after fermentation [41] but in the other one the amount was found to increase after alcoholic fermentation [34]. In the present study in Cava samples aged in contact with lees, NSER ranges from 0.3 μg to 2.3 μg (Table 2). Regarding the variation between categories, significant differences were found between base wine and the rest of categories (Figure 3). These were only shown in the biological ageing samples. Therefore, a tendency for their content to decrease as a function of ageing time could be observed, although the decrease did not significantly fit a correlation model with months of ageing. When comparing rosé and white commercial Cava it was found that, interestingly, the NSER concentration showed significant differences between these Cavas (Cava rosé 0.26 µg/L and white Cava 0.74 µg/L). The influence of grape variety has not been extensively studied, only in the case of Romanian wines it was found that the concentrations of melatonin and its precursors were higher in rosé wines [42]. Further studies are needed to clarify the role of skin maceration on NSER content.

Finally, 5MTL was detected in a range between 0.3 µg/L and 29.2 µg/L in only 22 samples of commercial and winery Cava (Table 2). As shown in Table 2, not all Cava samples reported this compound, as in some cases it was below the Limit of Detection (LOD) [22]. It could be seen that there is a certain tendency to reduce the amount of 5MTL the longer the Cava is aged. However, it was not possible to establish a correlation between the amount of this indole and Cava ageing. The presence of 5MTL in *S. cerevisiae* has been reported [43,44] but it was not clear the pathway that induced its formation [45]. Furthermore, it seemed that a higher presence of TRP did not increase the presence of this indole [43]. Therefore, the role of 5MTL in wines and lees remains to be clarified.

### 3.4. Multivariant Analysis

**Figure 6:**
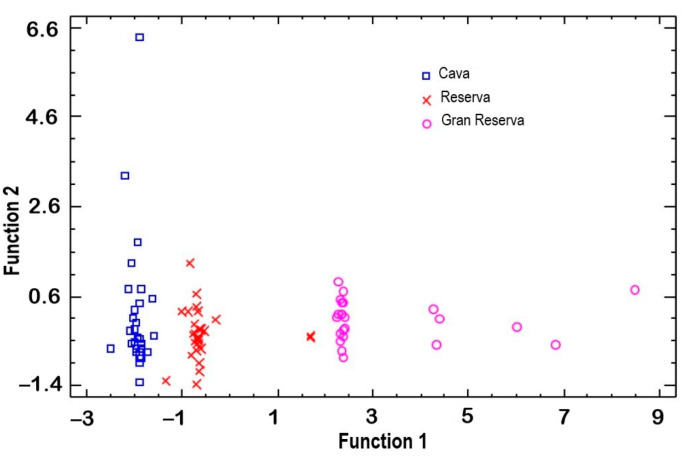
Discriminant analysis chart of biological ageing and commercial samples.

To confirm that the differences between the samples are attributable to biological aging with lees, a multivariate analysis was conducted. First, a discriminant analysis of the samples was conducted (Figure 6). This analysis showed that the samples were clearly separated by months of aging. It suggested that, despite the minor differences among the wineries, the aging factor had the greatest influence on the variability of the samples.

**Figure 7:**
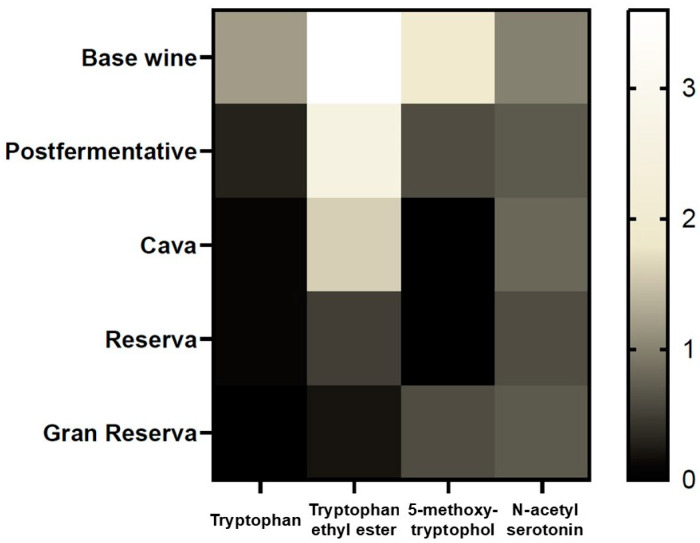
Heatmap of the mean of the indole content (TRP, TEE, 5MTL and NSER) in different categories of cava (*p* < 0.05). Largest values are shown in white; smallest values in black; baselines values in yellow. As the TRP is in much higher quantities it is represented on a smaller scale (1:1000).

In this sense, Figure 7 shows a heatmap to observe the overall variation of indole content as a function of ageing time. Lighter colors correspond to a higher abundance of the compound, while darker colors show a lower quantity of the estudied indole. As could be seen in the graph, the higher ageing time, the less content of TRP, TEE and 5MTL. That was in agreement with ANOVA that showed that TRP and TEE varied significantly between different categories of Cava and 5MTL showed a tendency to decrease (Table 2). Hence, this parameter could be used as an ageing marker in sparkling wines. Moreover, more studies are needed to clarify the role of lees in the amount of these compounds.

## 4. Conclusions

In conclusion, TRP, TEE, 5MTL and NSER were identified for the first time in biological ageing samples. Moreover, it was the first time that the latter two indoles were reported in wine. However, MEL and its isomers SER, 5-OHTRP, 5OHIA or 5MIA could not be detected, therefore, Cava is not a source of Melatonin.

Furthermore, TEE content is proposed as a possible biomarker of ageing in the Gran Reserva category. An amount lower than 0.56 μg TEE/L would help to define this category of sparkling wine and prevent fraud of these differentiated quality wines.

The most characteristic indoles of the base wines were TRP and TEE. They correlated positively and decreased with ageing time.5MTL and NSER showed a tendency to decrease during biological ageing, but no significant differences could be established. Therefore, it has been demonstrated that the content of indoles in Cava varies as a function of ageing time. Finally, according to our experimental results, it could be concluded that biological ageing plays a key role in determining the indole content in sparkling wine. However, further studies are needed to clarify the role of lees in the indole content.

## Figures and Tables

**Table 1:** Validation parameters of target indoles.

Compound	Calibration Curves ^a^			Range (µg/L) (n = 6)	LOD (µg/L)	Repeatability RSD % (n = 6)	Matrix Effects ^b^ (n = 6)	Accuracy % (n = 6)
	a	b	R					
5MIA	9823.1	1.50	0.9988	0–1000	0.61	7.5	+15.6%	97.9
5MTL	4277.2	0.80	0.9973		0.29	8.0	+12.9%	97.1
NSER	886.5	−0.10	0.9980		0.05	8.2	+0.5%	97.0
5OHIA	41,130.3	1.30	0.9982		1.46	9.3	−26.8%	99.2
TRP	3006.1	0.40	0.9999		0.13	5.9	−10.1%	98.5
MEL	288.5	−0.03	0.9935		0.02	4.8	+19.7%	98.3
5OHTRP	4072.7	0.90	0.9997		0.11	3.6	−38.8%	99.0
SER	25,841.8	2.70	0.9987		19.80	8.2	−14.2%	98.2
TEE	44.5	3.57	0.9953		0.01	6.4	−4.6%	101.0

^a^ [c]_S_ = (A_s_/A_I.S._) a ± b, where [c]_S_ = analyte concentration in sample, A_s_ = area of analyte, A_I.S._ = area of internal standard. ^b^ Mean response variation in the range of calibration in presence of matrix.

**Table 2:** Indoles content (µg/L) in different categories of Cava Wine (winery samples n = 29; commercial samples n = 45).

Concentration (µg/L) in Biological Ageing (Winery)															
Indoles	Base Wines (n = 6)			Post Fermentative Aging (n = 5)			Cava (n = 4)			Reserva (n = 6)			Gran Reserva (n = 8)		
	Max	Min	Average	Max	Min	Average	Max	Min	Average	Max	Min	Average	Max	Min	Average
5MTL	5.5	<LOD	1.9 ^a^	3.0	<LOD	<0.6 ^ab^	<LOD		<LOD ^b^	<LOD		<LOD ^b^	3.3	<LOD	0.4 ^b^
NSER	1.3	0.8	1.0 ^a^	0.9	0.6	0.7 ^b^	1.3	0.5	0.8 ^ab^	0.8	0.5	0.6 ^b^	0.7	0.5	0.7 ^b^
TRP	1680.4	250.3	1165.0 ^a^	855.1	84.9	326.8 ^b^	180.3	95.4	133.3 ^b^	144.7	30.3	77.2 ^b^	32.5	8.5	16.4 ^b^
TEE	5.2	2.3	3.6 ^a^	6.3	1.4	2.6 ^ab^	2.2	1.4	1.7 ^bc^	1.0	0.3	0.7 ^cd^	0.4	0.1	0.2 ^d^
**Concentration (µg/L) in commercial Cava**.															
							**Cava** **(n = 18)**			**Reserva** **(n = 15)**			**Gran reserva** **(n = 12)**		
							**Max**	**Min**	**Average**	**Max**	**Min**	**Average**	**Max**	**Min**	**Average**
5MTL							29.2	<LOQ	1.7 ^a^	5.4	<LOQ	0.9 ^a^	5.7	<LOQ	0.9 ^a^
NSER							2.3	0.3	0.8 ^a^	1.3	0.3	0.8 ^a^	1.1	0.4	0.7 ^a^
TRP							574.87	2.3	125.4 ^a^	179.5	2.9	66.4 ^ab^	128.1	5.9	38.8 ^b^
TEE							2.8	<LOD	0.7 ^a^	1.4	<LOD	0.4 ^ab^	0.6	0.1	0.3 ^b^

Small letters on the same line indicate statistically significant differences between means (*p* < 0.05). LOD = Limit of Detection; LOQ = Limit of Quantification.

## Data Availability

The original contributions presented in the study are included in the article, further inquiries can be directed to the corresponding author.
